# Scientific Items

**Published:** 1887-10

**Authors:** 


### SCIENTIFIC ITEMS.

**A Wise Wasp.**—While sitting, one summer day, at the side of the house on a platform which served as a piazza, but was roofed only by the branches of two large trees, something dropped upon my head and rolled into my lap, when I saw a large white bodied spider in the clutches of a small wasp. Hastily brushing these unceremonious visitors on to the floor, I watched to see if the wasp would succeed in flying away with his huge enemy. After a struggle the spider lay quiet, and the wasp ran around, seizing first one part, then another, but finally went away, as I supposed, for help. In about a quarter of an hour he returned, still alone, and began trying again, as I thought, to find some place by which he could seize the round body and carry it away. Again he departed without his spider. This time I watched him and saw him disappear at the edge of the lawn, under a pear tree, and, following, found him, after some searching, diligently at work with another wasp enlarging a hole in the ground, having already thrown out quite a little mound of earth. I was surprised, for I did not then know that any kind of wasp lived in the ground.

I returned to the piazza, and soon, when the wasp came back, I was convinced, by more careful watching, that he was measuring each part of the spider's body instead of trying to get hold of it. The antennæ seemed to be the organs mostly employed in this operation. When he went home again, I was before him, and saw him meet his co-worker, put his head close to his, and evidently informed him that the doorway was not yet big enough, for they fell busily at work enlarging it. Then more measuring, more digging, until, after three long hours, he returned, this time with his friend, and they carried away their prey and bestowed it in their underground home. Question for studious Agassizites: How many kinds of wasps are there, and how many have adopted the metric system ?—*The Owl.*

**How Arago Measured the Power of Steam.**—The experiments which were entered upon for the purpose of measuring the force of the vapor of water were very important and very dangerous: important, because the safe working of steam-engines was dependent upon correct measurements of the force, and because all the properties of heat had to be passed in review; and dangerous because they "imposed the task of confronting the unknown caprices of a formidable force There were but two men to accept it and conduct it to success: Arago, who never shrank from a duty; and Dulong, already maimed by an explosion, whose previous studies had admirably prepared him for the new work." A rude manometer was extemporized, and a boiler, far less staunch than the boilers of to-day, was set up, in which water was heated till the pressure was twenty-seven atmospheres. "They could not go further. At this extreme point it leaked at all the joints, and the steam escaped through the fissures with a hissing that was of bad omen. But the observers, though aware of the danger, silent and resigned, finished without accident the measurements which they had begun." Telling M. Jamin the story, which was written out as above from his dictation, Arago said: "Only one being of our company preserved his serenity and slept quitely; it it was Dulong's dog; they called him Omicron."—*P. S. News.*

**Electricity a Form of Matter.**—Mr. Carl Hering, writing to the *Electrical World*, says:

"It is a well-known fact that quantity of electricity measured in coulombs never is generated, never is consumed, and never does grow less, excepting leakage. The current flowing out of a lamp is exactly the same in quantity as that going into it; the same is true of motors and of generators, showing that electricity of itself is never consumed while doing work in a lamp or motor, it comes out in precisely the same quantity as it entered. A battery is not able to generate quantity or coulombs of electricity; all it is able to do is to take the quantity which pours in at one pole and send it out at the other pole with an increased pressure, or E.M.F. Electricity, therefore, is not merely force (or a form of energy), but matter. It is precisely analogous to water in a water circuit. The water is neither consumed nor generated. The pump merely increases the pressure of the water which flows in at one end. The water motor merely consumes the pressure, and converts it into mechanical work of another kind. It does not consume the water. The quantity of water, measured in units of quantity, is the same in all parts of a closed circuit of water," etc.—*Scientific American.*

**High Ballooning.**—The æronauts Mallet and Jovis made an ascent, August 13, in the balloon Horla, starting from the Lavillette gas works, Paris. Their object was to penetrate to the greatest height at which it is possible to live. After a few hours' voyage in the air the balloon descended, landing in the village Marche, Belgium. They reached an altitude of a little over four miles. This telegram has been received from M. Jovis:

"Victory! We attained an altitude of over 7,000 meters. We were obliged to descend for want of ballast. The conditions were excellent, except that M. Mallet fainted twice. The apparatus is intact."— *Scientific American.*

**Artesian Well at Galveston.**—An artesian well is being bored at Galveston. The city stands on a narrow sand spit, which fences off Galveston Bay from the Gulf of Mexico, and is surrounded by water, being at different places from two to forty miles from the mainland. It is therefore a peculiar place for an artesian well. So far a depth of 658 feet has been reached The following is the stratification passed through: Quicksand, 32 feet; blue clay, 17 feet; coarse sand, 26 feet; white clay, 107 feet; sea, mud, 57 feet; olive clay, 116 feet; sea mud, 130 feet; blue clay, 29 feet; sea mud, 11 feet; blue clay, 147 feet; total, 658 feet. At a depth of 600 feet several palmetto logs were passed through. At present a 9 inch tube is being sunk.—*Ib.*

